# p53 predominantly regulates IL-6 production and suppresses synovial inflammation in fibroblast-like synoviocytes and adjuvant-induced arthritis

**DOI:** 10.1186/s13075-016-1161-4

**Published:** 2016-11-24

**Authors:** Ting Zhang, Huihua Li, Juan Shi, Sha Li, Muyuan Li, Lei Zhang, Leting Zheng, Dexian Zheng, Fulin Tang, Xuan Zhang, Fengchun Zhang, Xin You

**Affiliations:** 1 Department of Rheumatology and Clinical Immunology, Peking Union Medical College Hospital, Chinese Academy of Medical Sciences & Peking Union Medical College, Key Laboratory of Rheumatology and Clinical Immunology, Ministry of Education, Beijing, 100730 China; 2 Basic Science Institute, Chinese Academy of Medical Sciences & Peking Union Medical College, Beijing, 100730 China

**Keywords:** Rheumatoid arthritis, p53, Fibroblast-like synoviocytes, Interleukin-6, Signal transduction

## Abstract

**Background:**

Dominant-negative somatic mutations of p53 has been identified in the synovium of patients with rheumatoid arthritis (RA), in which interleukin (IL)-6 has been established as a pivotal inflammatory cytokine. The aim of this study was to clarify the significance of p53 in the longstanding inflammation in RA by modulating IL-6.

**Methods:**

We established adjuvant-induced arthritis (AIA) in Lewis rats and treated them with p53 activator, and then analyzed the histopathology of the synovium and IL-6 expression. Human fibroblast-like synoviocytes (FLS) were cultured and transfected with p53-siRNA or transduced with adenovirus (Ad)-p53, and then assessed with MTT, TUNEL staining, and luciferase assay. IL-1β, tumor necrosis factor (TNF)-α and IL-17 were used to stimulate FLS, and subsequent IL-6 expression as well as relevant signal pathways were explored.

**Results:**

p53 significantly reduced synovitis as well as the IL-6 level in the AIA rats. It controlled cell cycle arrest and proliferation, but not apoptosis. Proinflammatory cytokines inhibited p53 expression in FLS, while p53 significantly suppressed the production of IL-6. Furthermore, IL-6 expression in p53-deficient FLS was profoundly reduced by NF-kappaB, p38, JNK, and ERK inhibitors.

**Conclusion:**

Our findings reveal a novel function of p53 in controlling inflammatory responses and suggest that p53 abnormalities in RA could sustain and accelerate synovial inflammation mainly through IL-6. p53 may be a key modulator of IL-6 in the synovium and plays a pivotal role in suppressing inflammation by interaction with the signal pathways in RA-FLS. Interfering with the p53 pathway could therefore be an effective strategy to treat RA.

## Background

Rheumatoid arthritis (RA) is a chronic progressive polyarthritis that is characterized by synovial inflammation and pannus formation, which leads to destruction of cartilage and bone resulting in joint deformities and disability [1]. The normal synovium consists of an intimal lining with one to two cell layers, which expand in RA in part due to increased numbers of fibroblast-like synoviocytes (FLS). FLS are the major source of proinflammatory cytokines and matrix degrading enzymes that are responsible for joint inflammation and destruction [2]. The aggressive properties of RA-FLS are similar to neoplasms, with the underlying mechanisms that include mutations of tumor suppressor genes such as p53 [3–6].

A dominant negative mutation of p53 in RA synovium and FLS has been associated with its dysfunction and interleukin (IL)-6 production [7]. For many years, this has been traditionally attributed to insufficient synoviocyte apoptosis in RA. While the exact cause of this is not entirely known, the presence of microsatellite instability manifested by damaged DNA and decreased expression of DNA mismatch repair protein in rheumatoid synovium could lead to the point mutation of p53 [8]. Animal experiments showed increased disease severity in p53–/– mice compared to p53+/+ mice in the collagen-induced arthritis (CIA) model, confirming the significance of p53 in inflammatory arthritis [9]. However, adenovirus (Ad)-mediated p53 is not capable of inducing apoptosis in RA-FLS in vitro. Therefore, we suspect that this remarkable gene may be involved in more than just apoptosis, and may instead play distinctive roles in inflammatory arthritis.

Our preliminary research revealed that the p53 activator (Nutlin) ameliorated adjuvant-induced arthritis (AIA) in Lewis rats, although the effect of p53 did not appear to be mediated by inducing apoptosis in FLS, and p53-overexpressed FLS arrested at phase G1. Histologic analysis of the synovium demonstrated dramatic decreases in infiltration of inflammatory cells after induction of p53 in vivo. These results raised further questions about the role of p53 in synovitis since p53-mediated cell cycle arrest might not account for its effect. We now address a novel, crucial role of p53 in the regulation of synovium inflammation.

## Methods

### Induction of adjuvant-induced arthritis in rats

Lewis rats were purchased from the animal center in Peking Union Medical College (PUMC) and were maintained under specific pathogen-free conditions at the PUMC Hospital. All animal procedures were performed with the approval of the Institutional Animal Care and Use Committee of the PUMC Hospital.

Lewis rats, 8-week-old females, were given intracutaneous (i.c.) injections of *Mycobacterium tuberculosis* (0.1 mg emulsified in 100 μl of paraffin oil; Chondrex Inc., Japan) at the right footpad of the rear paw as described previously [10]. On day 0 they were randomly divided into three groups: a Nutlin-treatment group as well as two control groups designed to receive dimethyl sulfoxide (DMSO) or phosphate-buffered saline (PBS) (*n* = 6 for each). From day 8 to day 12 after induction, rats were administered with Nutlin-3 (250 mg/kg in DMSO per day, intraperitoneally), DMSO, or PBS.

The severity of arthritis was assessed by scoring arthritis every 3 days and measuring the volume of hind paw swelling with water displacement on day 8 and day 21 after immunization. The rats then were sacrificed and the serum and ankle joint homogenates were prepared for IL-6 detection. Tissues harvested from dissected ankles were fixed in 10% buffered formalin and decalcified, embedded, sectioned, and stained with hematoxylin and eosin (H&E) to grade inflammatory and bone destructive changes. Sections were randomly analyzed. All areas of each section were independently examined by microscopy by two observers.

### Human fibroblast-like synoviocyte culture

Synovial tissues were obtained from patients with RA and osteoarthritis following joint replacement surgery. The diagnosis of RA met the American College of Rheumatology 1987 revised criteria [11]. The protocol was approved by the PUMC Hospital Human Subjects Research Protection Program. FLS were isolated from individual tissues with 1 mg/ml collagenase and cultured in DMEM supplemented with 10% fetal calf serum, penicillin, streptomycin, and l-glutamine. Cell lines were used from the third to ninth passage on the basis of vascular cell adhesion molecule (VCAM)-1 and CD55 expression, when they were a homogeneous population of fibroblast-like cells [12].

### Antibodies

Affinity-purified mouse monoclonal anti-p53 for Western blotting and rabbit polyclonal antibodies against p21 were purchased from Santa Cruz Biotechnology (Santa Cruz, USA). Anti-(phospho-) p38, anti-(phospho-) JNK, anti-(phospho-) ERK, anti-(phospho-) IκBα and anti-mouse and anti-rabbit IgG secondary antibodies were purchased from Cell Signaling Technology, Inc. (Beverly, MA, USA).

### Cell transfections

Scrambled RNA and p53 siRNA were purchased from Dharmacon Research, Inc. (Lafayette, CO, USA). Cells were transfected with the use of the Amaxa Human Dermal Fibroblast Nucleofactor kit (Amaxa Biosystem, Germany) with program U-23 for human FLS. Cells (2 × 10^5^ to 10^6^) were transfected with siRNAs in each reaction.

### Transduction of adenovirus-p53

To deliver p53 into FLS, recombinant human Ad-p53 was added to serum-free supernatant; multiplicity of infection (MOI) was 100. The plate was shaken every 10 min to ensure full contact of adenovirus with FLS. The supernatant was then replaced with serum-supplemented DMEM 3 h later.

### MTT assay

To measure cell viability, FLS were trypsinized and washed, and approximately 1 × 10^4^ cells were seeded into each well of 96-well culture plates. The MTT working solution was 0.5 mg/ml MTT in PBS for 4 h at 37 °C, then replaced with DMSO. The plate was analyzed on a spectrophotometric plate reader at a test wavelength of 570 nm and a reference wavelength of 630 nm.

### TUNEL staining

To detect fragmented DNA in FLS and synovium, a TUNEL kit, DNA nick end labeling (R&D Company, USA), was used as directed. Nuclei positive for TUNEL staining were stained with green fluorescence by the reaction. The level of positive staining was determined by visual observation through fluorescence microscopy.

### Luciferase assay

Cells were transfected with 5 μg of a Bax-promoter-luciferase construct (bax-luc), or empty vector (pCI) at day 3 post-siRNA transfection, and harvested 24 h and 60 h after plasmid transfection. Results were normalized to co-transfected β-galactosidase expression as relative luciferase units (RLU).

### Stimulation of FLS with inflammatory cytokines

Inflammatory cytokines, including tumor necrosis factor (TNF)-α (R&D), IL-1β (R&D), and IL-17 (R&D) were added alone or in combination to the supernatants of DMEM for incubation with FLS. The final concentrations of these cytokines and length of incubation period varied for different research targets.

### Inhibition of signal pathways

Cultured FLS were incubated with inhibitors of p38, JNK, ERK, and NF-kB for 30 min before IL-1β were added. The inhibitors were SB203508 (Sigma, USA), SP600125 (Calbiochem, Merck), U0126 (Calbiochem, Merck), and PTDC (Sigma, USA), respectively. The final concentration of IL-1β was 0.1 ng/ml. Supernatant was recovered 24 h later for detection of IL-6 with an ELISA kit (Dakewe, China).

### Western blot analysis

Cultured FLS were washed with PBS, and protein was extracted with lysis buffer (50 mM HEPES pH 8.0, 150 mM NaCl, 1% Triton X-100, 10% glycerol, 1 mM MgCl_2_, 1.5 mM EDTA, 20 mM β-glycerophosphate, 50 mM NaF, 1 mM Na_3_VO_4_, 10 μg/ml aprotonin, 1 μM pepstatin A, 1 mM phenylmethylsulphonyl fluoride). The protein concentrations were determined with the DC protein assay kit (Bio-Rad, Hercules, CA, USA). Whole cell lysates containing 50 μg of protein were fractionated by 12% SDS-PAGE and transferred to a nitrocellulose membrane. The membrane was blocked with Tris-buffered saline plus 0.1% Tween 20 (TBST) containing 5% non-fat milk for 1 h at room temperature followed by incubation overnight with the appropriate antibody at 4 °C. The membrane was washed three times and incubated with horse radish peroxidase-conjugated secondary antibody for 1 h. Immunoreactive protein was detected by chemiluminescence with Kodak X-AR film (Eastman Kodak, Rochester, NY, USA).

### ELISA

ELISA kits were used to detect rat IL-6 (R&D, USA) and human IL-6 (Dakewe, China) or MMP-1 (R&D, USA) in recovered supernatants. Procedures were carried out following the instructions enclosed in the kits.

### CBA kit to detect inflammatory cytokines

Human FLS with different p53 status were cultured and incubated with IL-1β or TNFα. After 24 h the supernatant was harvested, and IL-17, TNFα, IL-2, IL-4, IL-10, and IFN-γ were detected using a CBA kit (BD Bioscience).

### Cell proliferation assay

Alamar Blue assays incorporate a fluorimetric/colorimetric growth indicator based on the detection of metabolic activity. FLS (3 × 10^3^) were plated into a 96-well plate after siRNA transfection. At various time points, medium was replaced by DMEM without phenol red supplemented with 10% Alamar Blue. After incubation for 4 h at 37 °C, fluorescence was measured with a microplate reader at an excitation wavelength of 530 nm and an emission wavelength of 590 nm. The number of cells was expressed as relative fluorescence units.

### Statistical analysis

Data are expressed as means ± SEM. Statistics were performed with Student’s *t* test, one-way analysis of variance and repeated-measures analysis of variance. A comparison was considered significant at *p* < 0.05.

## Results

### P53 reduced arthritis and synovitis in the AIA rats

**Fig. 1:**
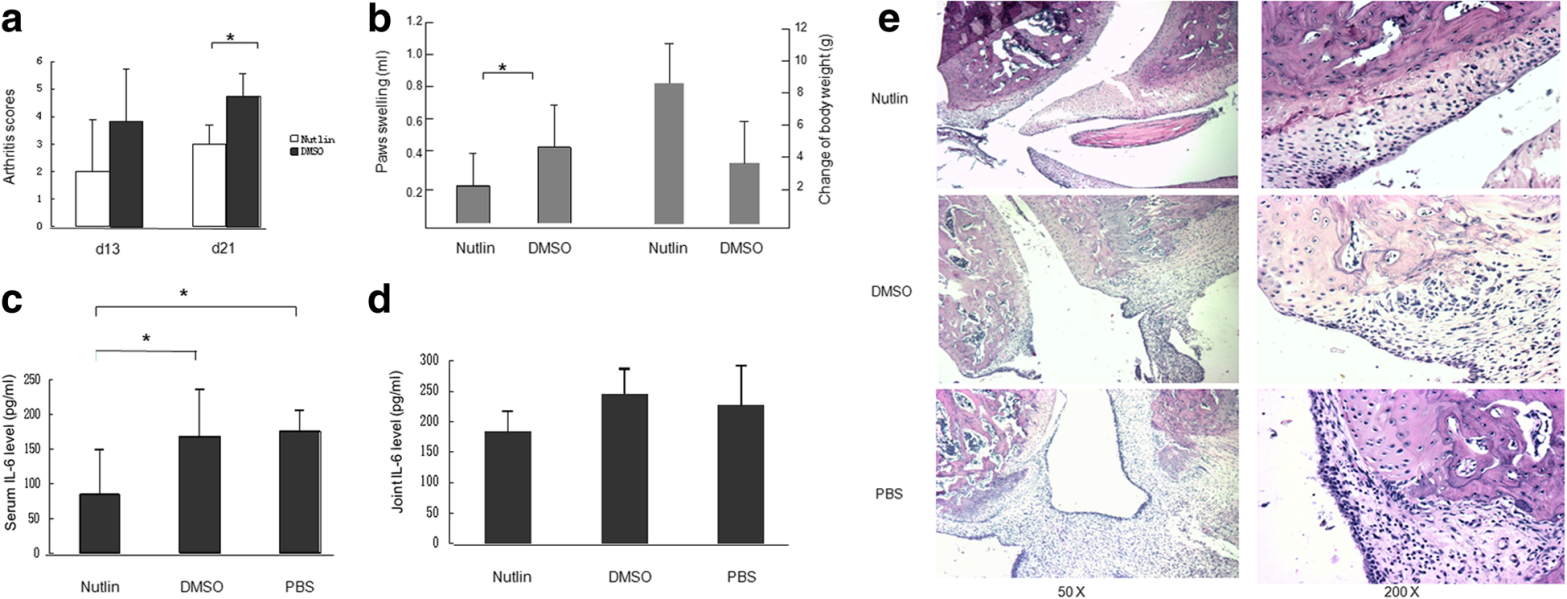
Nutlin-3 attenuated arthritis and decreased IL-6 production in Lewis AIA rats. Lewis rats were immunized with complete Freund’s adjuvant, and received Nutlin-3, dimethyl sulfoxide (*DMSO*), or phosphate-buffered saline (*PBS*) from day 8 to day 12 after immunization. Arthritis scores were assessed every 3 days. **a** The mean arthritis scores. Nutlin significantly decreased arthritis, compared to DMSO (*p* = 0.16 and 0.02, respectively, on day (d)13 and day 21). **b** Severity of inflammation was calculated by subtracting the volume of hind paw swelling between day 21 and day 8 after immunization (*p* = 0.04, Nutlin versus DMSO) and the body weight of the Nutlin group has a tendency to increase (*p* = 0.14, Nutlin versus DMSO). **c** Interleukin-6 (*IL-6*) levels in the serum of the Nutlin group were significantly decreased compared to those in the DMSO and PBS groups (*p* = 0.01 and 0.04, respectively). **d** IL-6 level in the joint (*p* = 0.09, Nutlin versus DMSO; p = 0.19, Nutlin versus PBS). **e** Nutlin-3 decreased infiltration of inflammatory cells into the synovial tissue and bone erosion in AIA rats. Infiltration of inflammatory cells into the synovial tissue and pannus formation were observed in DMSO and PBS group, but not in the Nutlin group. *p < 0.01

To test the efficacy of p53 in treating inflammatory arthritis, AIA in the Lewis rat model was used. Intraperitoneal injection of Nutlin-3, a p53 activator, ameliorated arthritis dramatically, including a significant decrease in arthritis scores and joint swelling relative to the vehicle-treated group (*p* < 0.05; Fig. 1a and b). There was a trend for increased body weight in the Nutlin group compared to that in the vehicle group (Fig. 1b). Histopathologic analysis indicated that the synovium in Nutlin-treated rats was less inflamed than those treated with vehicle or PBS (Fig. 1e).

High levels of local IL-6 were measured in the serum and joint homogenates using ELISA (Fig. 1c and d). Nutlin significantly reduced IL-6 production in the serum relative to the DMSO and PBS groups (*p* < 0.05). Although there was no significant difference in joint IL-6 levels between the Nutlin and vehicle groups or the Nutlin and PBS groups (*p* > 0.05), a tendency for decreased IL-6 with Nutlin was observed.

### P53 controlled cell cycle arrest and proliferation, but not apoptosis, in FLS

**Fig. 2:**
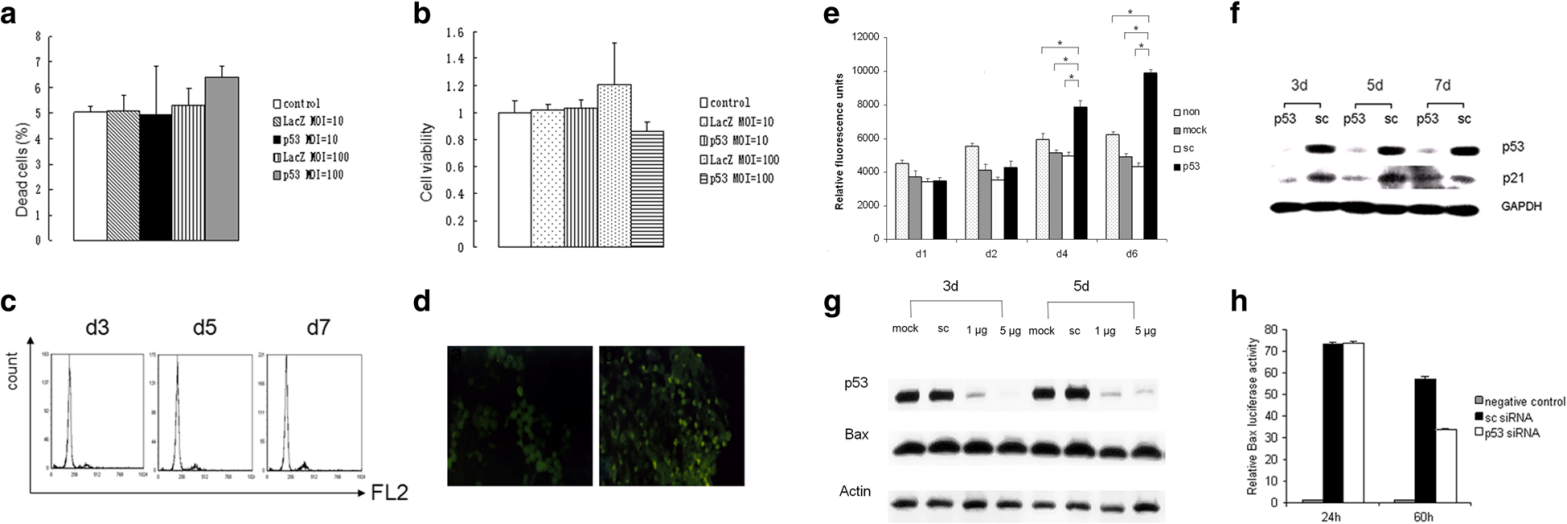
p53 did not induce apoptosis in FLS or the synovium (**a–d**), but did have control over cell growth (**e**–**h**). **a** Trypan blue staining 3 days following transfection with Ad-LacZ and Ad-p53 at different multiplicity of infection (*MOI*). **b** MTT assay 3 days following transfection with Ad-LacZ and Ad-p53 at different MOI. **c** Cell cycle in FLS following transfection with Ad-p53 (MOI = 100) on day (d)3, d5, and d7. **d** TUNEL staining on slides of collected FLS (*left*) and synovium 3 days following Ad-p53 treatment (*right*). **e** Deletion of p53 in FLS increased cell growth as measured by Alamar blue staining. *,p<0.05 **f** Deletion of p53 in FLS contribute to the lower expression of p21 thereafter. **g** Bax expression following knockdown of p53 was analyzed by Western blot. **h** Effects of p53 expression on bax promotor activity in FLS as measured by luciferase assay. non, blank control. mock, mock transfection. sc, scrambled RNA control

To address the mechanism of p53 on the therapy of inflammatory arthritis, we first delivered p53 to FLS using adenovirus in vitro to identify the possible effects of p53 as a tumor suppressor, including regulation of the cell cycle and induction of apoptosis. As previously noted, ectopic expression of p53 did not induce apoptosis in FLS. There were no significant differences between the Ad-p53 treated group and the Ad-LacZ treated group when counting dead cells with trypan blue staining (Fig. 2a) or when measuring cell viability with the MTT assay (Fig. 2b). Cell cycle analysis by flow cytometry indicated that the p53-overexpressed FLS arrested at phase G1 following transfection with Ad-p53 on days 3, 5, and 7, respectively (Fig. 2c). TUNEL staining demonstrated less DNA fragmentation in Ad-p53 transfected FLS in vitro (Fig. 2d, left panel) and in synovium from the Ad-p53 treatment group in vivo (Fig. 2d, right panel).

Conversely, p53 knockdown significantly promoted cell growth as measured by Alamar blue staining on day 4 and day 6 following the p53-siRNA transfection in FLS (Fig. 2e). As expected, p21, a downstream protein of p53, was also reduced following the suppression of p53 in FLS (Fig. 2f). However, another classical downstream factor of p53, Bax, was not changed following the alteration of p53 status in FLS both at translational and transcriptional levels detected by Western blot (Fig. 2g) and luciferase assay (Fig. 2h), consistent with the finding that cell survival was not affected by ectopic expression of p53.

### Proinflammatory cytokines inhibited p53 expression in FLS

**Fig. 3:**
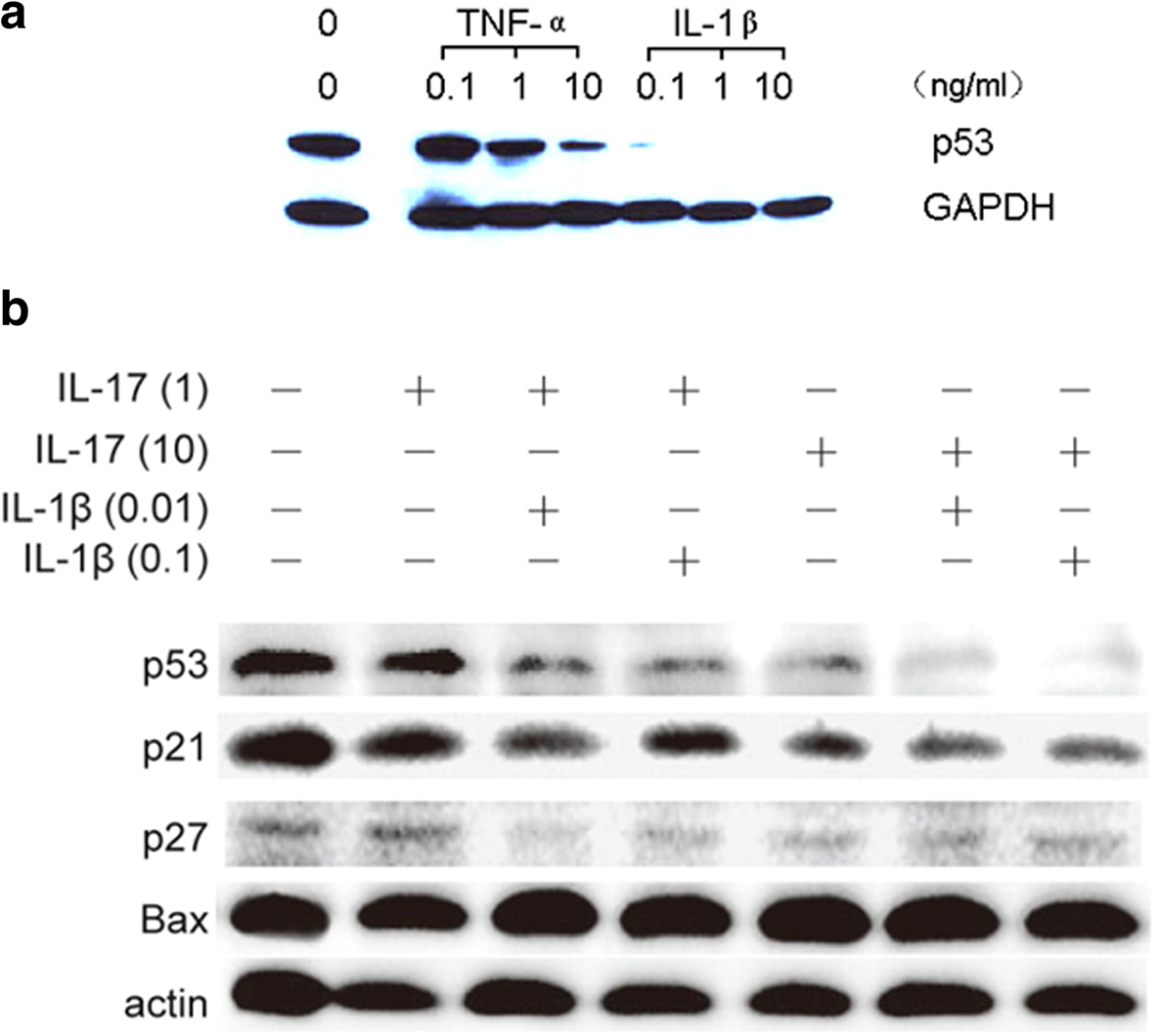
Inflammatory cytokines inhibited expression of p53 in FLS in a dose-dependent manner. **a** p53 expression was reduced in FLS after the stimulation with TNF-α and IL-1β at 0.1, 1.0, and 10 ng/ml, respectively. **b** IL-1β and IL-17 synergistically inhibited expression of p53 and its related proteins p21 and p27, but not Bax. *IL* interleukin, *TNF* tumor necrosis factor

Based on the above results, we then investigated the role of p53 in the process of inflammation in RA. To mimic the inflammatory environment in the arthritis joint, TNF-α, IL-1β, and IL-17 were used to treat FLS. p53 expression was decreased after 24 h of stimulation at a dose-dependent manner in both the TNF-α and IL-1β groups. IL-1β at 0.1 ng/ml dramatically reduced expression of p53, even to a greater extent than TNF-α at 10 ng/ml (Fig. 3a). Then FLS were incubated with different combinations of IL-1β at 0.01 or 0.1 ng/ml and IL-17 at 1 ng/ml or 10 ng/ml. IL-17 alone or in combination with IL-1β also inhibited p53 expression in a dose-dependent manner (Fig. 3b). Decreased expression of p21, a downstream protein of p53, further confirmed the inhibition of p53, but Bax was not affected, which was also consistent with our previous results with p53 (Fig. 2g).

### p53 significantly suppressed the production of IL-6 in FLS

**Fig. 4:**
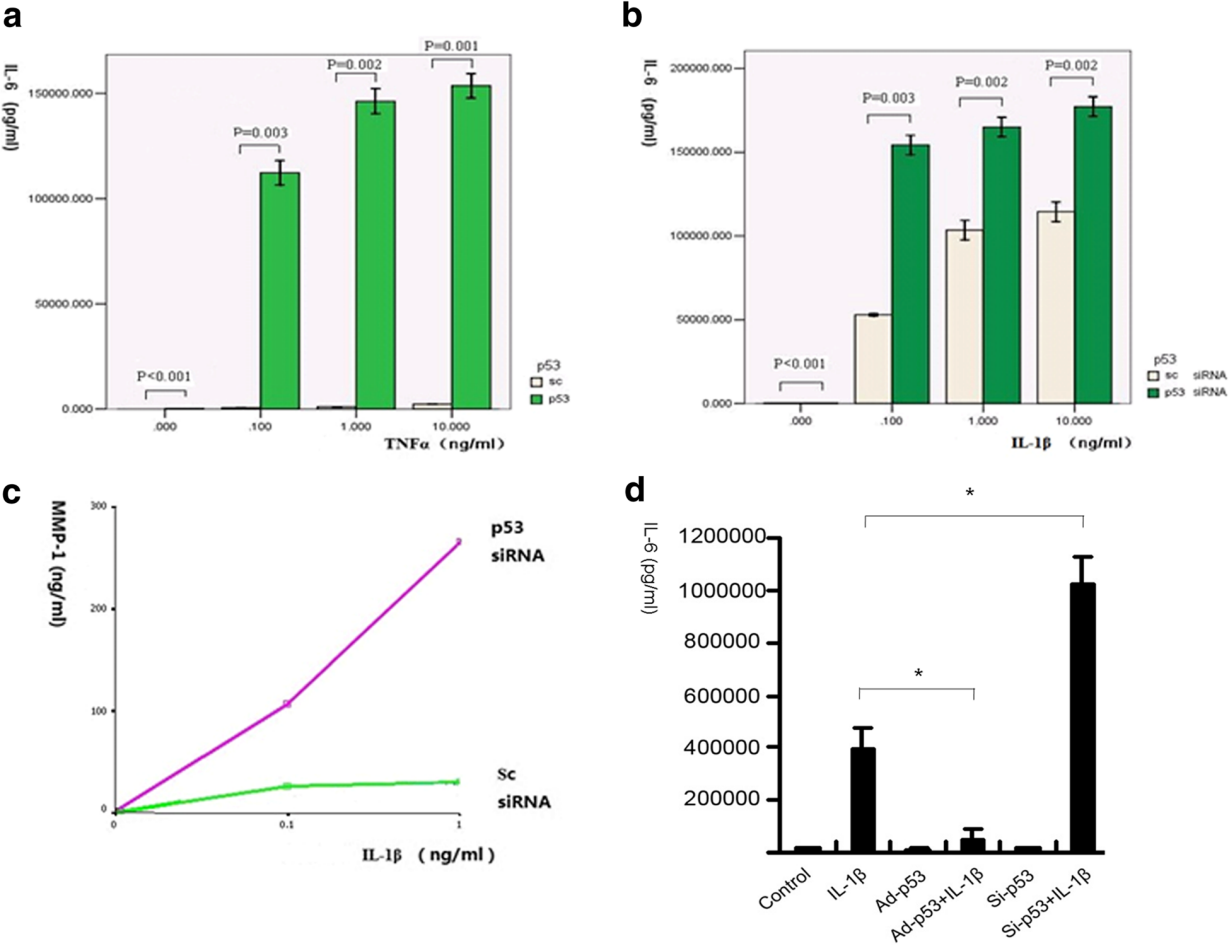
p53 modulated the production of proinflammatory factors in FLS. **a** Expression of IL-6 in FLS was efficiently inhibited following initial transfection of p53 siRNA. After incubation with TNF-α for 24 h, IL-6 greatly increased in the p53-deleted FLS. **b, c** Inhibition of p53 resulted in more production of IL-6 as well as MMP-1 after 24 h of incubation with IL-1β. **d** p53 suppressed the production of IL-6 in FLS following the stimulation of IL-1β. *Ad* adenovirus, *IL* interleukin, *MMP* matrix metalloproteinase, *sc* scrambled RNA control, *TNF* tumor necrosis factor

We then used p53 siRNA to reduce p53 expression by as much as 98% in FLS (Fig. 2f). On the third day, p53-deficient FLS were incubated with TNF-α or IL-1β for 24 h. IL-6 and MMP-1 concentrations in the supernatant were measured by ELISA. Both TNF-α and IL-1β increased IL-6 secretion in FLS in a dose-dependent manner, and p53 deficiency significantly amplified the increase in IL-6 secretion following either TNF-α or IL-1β stimulation (*p* ≤ 0.001). The amount of IL-6 production by FLS at a TNF-α concentration of 10 ng/ml was similar to that of IL-1β at 0.1 ng/ml, indicating that IL-1β had a stronger effect on stimulating IL-6 secretion than TNF-α (Fig. 4a and b), but the production of other proinflammatory cytokines by FLS following TNF-α or IL-1β stimulation in different p53 statuses were undetectable, including IL-17, TNFα, IL-1β, IL-4, IL-10, and IFN-γ. Furthermore, IL-1β at 0.1 ng/ml and 1 ng/ml also significantly increased MMP-1 production in p53 siRNA-transfected FLS, compared with scrambled siRNA-transfected FLS (*p* < 0.05; Fig. 4c).

To confirm the above results, we also treated the Ad-p53 transfected FLS with IL-1β and found that overexpression of p53 significantly inhibited IL-6 production (*p* < 0.001; Fig. 4d).

### p53 inhibited the activation of phosphorylated proteins in different signal pathways to suppress inflammation in FLS

**Fig. 5:**
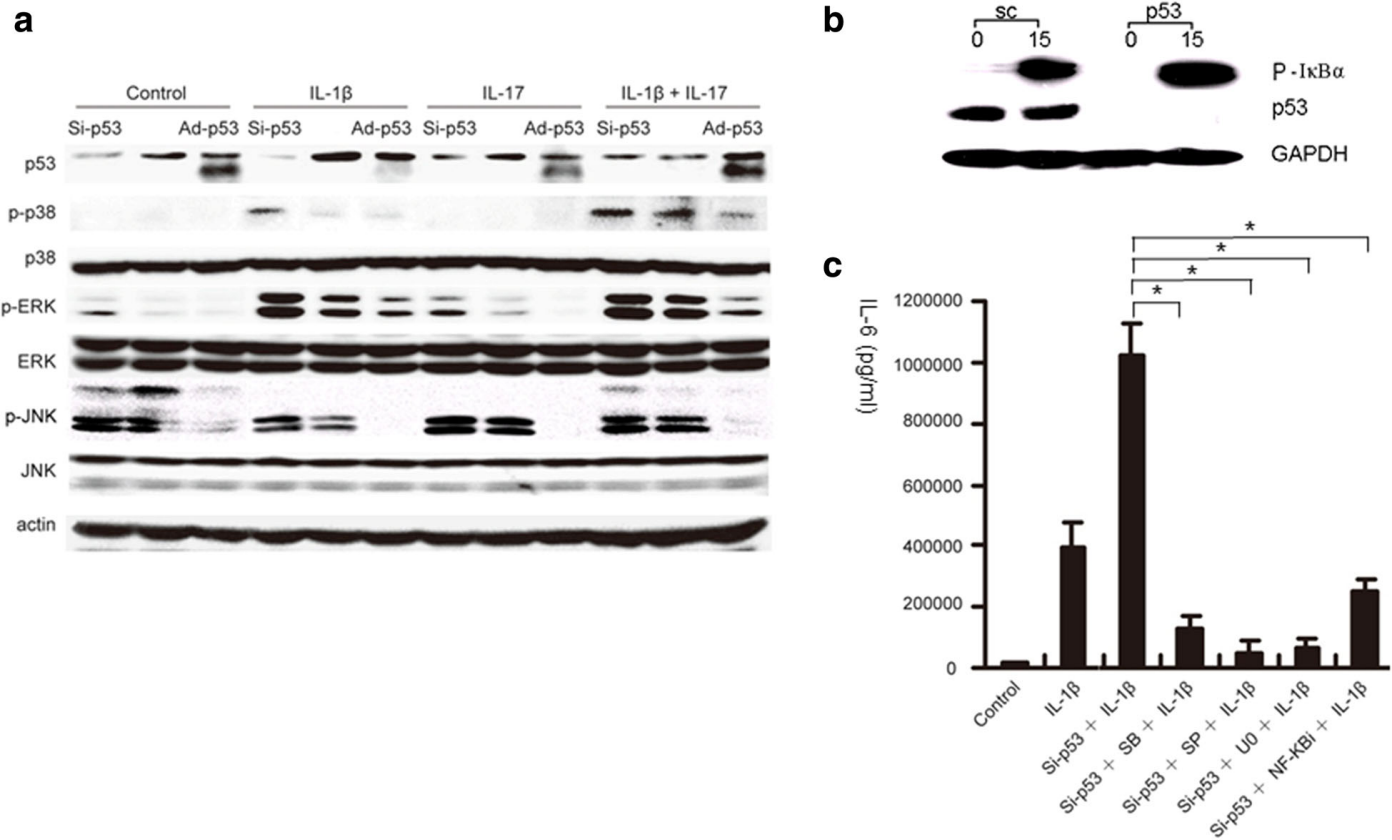
p53 in FLS strongly controls different signal pathways to suppress proinflammatory cytokine production. **a, b** Different p53 status in FLS, including deficient p53, wild-type p53, and overexpressed p53, regulated the phosphorylation of p38, JNK, and ERK, as well as IkBα in the FLS. **c** p53 insufficiency in FLS promoted the inflammatory cytokine production, which was significantly blocked by different signal transduction inhibitors, similar to the effects of overexpressed p53 on the suppression of IL-6 production in FLS (Fig. 4d; Ad-p53 + IL-1β). *Ad* adenovirus, *IL* interleukin, *TNF* tumor necrosis factor

To address the mechanism of p53 controlling IL-6 production in the synovium, the upstream signal transduction pathways in the inflammatory process of RA were investigated. FLS with varying p53 status were prepared, including p53 knockdown, wild-type (wt) p53, and p53 overexpressed. Cells were then stimulated with IL-1β for 15 min and evaluated by Western blot analysis for NF-κB and MAPK pathways (Fig. 5a and b). p53 deficiency significantly augmented phosphorylation of IkBα, p38, JNK, and ERK in the FLS, while overexpression of p53 significantly reduced phosphorylation of the same proteins. The results strongly indicated that p53 inhibited inflammation-related signal transduction in FLS.

To confirm the above results, indicating the role of p53 in different signal pathways, we treated FLS with SB203580 for the inhibition of p38, SP600125 for JNK, U0126 for ERK, and PTDC for NF-κB, respectively, 1 h earlier than incubating with IL-1β (0.1 ng/ml), and then measured the IL-6 levels in the supernatants. In p53-deleted FLS, production of IL-6 was significantly blocked by the signal transduction inhibitors (Fig. 5c). The effect of each of the inhibitors on the suppression of IL-6 in p53-deficient FLS was similar to that of overexpression of p53 on the reduction of IL-6 in Ad-p53 transfected FLS (Fig. 4d). The results might suggest that the intrinsic mechanism of p53 inhibiting inflammation is via profoundly suppressing signal transduction pathways.

## Discussion

RA is a chronic inflammatory disease with synovitis as its primary pathologic change. Nutlin-3A (a p53 activator), showing promising efficacy in the therapy of the AIA model through activation of p53, inhibits arthritic synovitis in vivo. In RA-FLS, expression of p53 was suppressed by cytokines, including IL-1β, TNF-α and IL-17, leading to subsequent stimulation of IL-6 production by activating the NF-κB and MAPK pathways.

NF-κB is one central component involved in all inflammation-associated conditions and all stages of inflammation. In our experiment, the suppression of p53 by inflammatory cytokines resulted in NF-κB activation. This phenomenon was consistently found in cancer. The majority of tumors with constitutive expression of NF-κB are p53 deficient. Other studies have revealed that NF-κB upregulates the levels of the major p53 inhibitor (Mdm2) [13–15], phosphorylates p53 through IKKβ [16], and induces anti-apoptotic factors to neutralize the pro-apoptotic effects of p53 [17]. Therefore, activation of NF-κB shutting down p53 function and responses in some cancer cells may also apply to chronic inflammation in RA synovium, leading to long-lasting p53 deficiency with mutant p53.

p53 also appears to act as a repressor of NF-κB in vivo since several reports using p53-null mice and wild-type mice demonstrated that p53 reduced inflammation in animal models, including CIA [9], experimental autoimmune encephalitis [18], and inflammatory lung injury caused by bleomycin [19, 20]. p53, responding to intrinsic stresses, limits the consequences of stress by initiating cell death, senescence, or cell cycle arrest, and promotes metabolic patterns in the cell to favor oxidative phosphorylation, while NF-κB promotes cell division. On activation of one of these transcription factors, the other is inactivated [21]. Some research groups suggest that their reciprocal inhibition is due to the common use of a limiting pool of co-activators [22–24]. Others reported a p53-mediated increase in IκB expression through downregulation of IκB kinase, which sequesters NF-κB signaling in the cytoplasm [25–27]. In addition, a direct association between p53 and p65, which mutually compromises their activation potential, has also been reported [28].

Besides NF-κB, the phosphorylation of all three main kinases in the MAPK pathway is also upregulated by p53 deficiency in FLS, including the stress-activated protein kinase (SAPK/c-Jun N-terminal protein kinase (JNK)), the p38 mitogen-activated protein kinase (p38), and the extracellular signal-related kinase (ERK). Recent reports of MAPK inhibitory phosphorylases as p53 targets have provided a more complex and comprehensive picture of how p53 functionally interacts with the MAPK signaling pathway. Activation of phosphatases by p53 negatively regulates MAPK-dependent signaling, including Wip1, MKP1, PAC, and DUSP5 [29–32]. These four phosphatases are capable of inactivating the MAPK signaling pathways through dephosphorylation [33]. Our research on the relationship between p53 and MAPK as well as NF-κB signaling pathways in synovial fibroblasts were consistent with those former results from cancer cells.

IL-6 is a key cytokine involved in the pathogenesis of RA, and recent research has demonstrated its central role in regulating the balance between IL-17 producing T helper (TH17) cells and regulatory T (Treg) cells. TH17 cells are potent inducers of IL-6, IL-8, MMP-1, and MMP-3 expression by the FLS [34], acting as the primary proinflammatory effector cells involved in RA [35]. Treg cells have an anti-inflammatory role and maintain tolerance to self-components [36], restraining excessive effector T-cell responses [35]. In the peripheral blood of patients with active RA, levels of TH17 cells are increased while Treg cells decreased [37]. IL-6 has been recognized as a regulator of the TH17/Treg balance. IL-6 induces the development of TH17 cells from naïve T cells together with transforming growth factor (TGF)-β, while inhibiting TGF-β promotes Treg differentiation [35]. In RA synovium, synovial fibroblast-derived IL-6 has a crucial role in the conversion of Foxp3 + CD4+ cells to TH17 cells. These TH17 cells are more potent osteoclastogenic T cells than naive CD4+ T cell-derived TH17 cells [38]. Treatment with the anti-IL-6 receptor antibody tocilizumab induces a significant decrease in the percentage of TH17 cells and an increase in the percentage of Treg cells in RA patients, resulting in a correction of the TH17/Treg cell imbalance [37, 39]. Although controversial results suggest unaffected TH17 cells with upregulated Treg cells after anti-IL-6 receptor treatment, there is a trend that TH17 cells are decreased [40–42]. So, combined with our results, we assume that p53 deficiency in RA promotes arthritic inflammation through upregulation of IL-6, which exerts its pivotal role by converting Tregs to TH17 cells. This has also been supported by the results from Ningli Li et al, demonstrating that Cyr61, an extracellular matrix protein regulated by p53, promotes IL-6 secretion as well as subsequent TH17 differentiation [43–45].

Thus, we hypothesize that p53 plays a key role in inhibiting synovial inflammation; the inflammation of the synovium in RA significantly inhibits p53 expression or leads to p53 mutation and dysfunction. In turn, suppressed p53 in synovial fibroblasts facilitates the activation of NF-κB and MAPK pathways, resulting in increased secretion of IL-6 which further inhibits p53 probably through TH17/Treg disturbance. This vicious feedback loop contributes to the perpetuation of inflammation and joint destruction in RA.

Besides involvement in synovial inflammation, our study also revealed that p53 controlled cell growth but failed to induce apoptosis in FLS. Increased cell growth following knockout of p53 in FLS is well explained by decreased expression of p21 and promoted phosphorylation of ERK. ERK is known to regulate cellular proliferation and differentiation [46], and p21 is tightly controlled by p53, through which p21 mediates the p53-dependent cell cycle arrest in response to a variety of stress stimuli [47]. Inhibition of p53 in FLS leads to reduced p21, which then affects both cell cycles and inflammation [48].

We also measured Bax, the Bcl-2-associated X protein, which is well recognized in p53-mediated apoptosis such as PUMA (p53-upregulated modulator of apoptosis) [49, 50]. However, in RA-FLS, PUMA-induced apoptosis does not require p53 [51]. Expression and function of Bax also does not change when p53 is deleted. This result is different from a previous report in which mutant p53 reduced Bax level in dermal fibroblasts [7]. In that paper, chemical transfection with mutant p53 was used rather than the siRNA technique. Although Bax is a key pro-apoptotic effector of p53 in most of the cases, it is possible that p53 does not control Bax in the specific tissue of synovium, and it gives us a reasonable explanation why p53 cannot induce apoptosis in FLS, suggesting that there might be other mechanisms for aberrant apoptosis induced by p53 in RA synovium.

Besides being deficient in FLS, p53 is also recognized to be reduced in peripheral blood mononuclear cells from patients with RA. The study by Maas et al. suggests that defects in the expression of p53 mRNA in the lymphocytes leads to severe defects in apoptosis in patients with RA [52]. Combined with our research, further investigation is needed to determine the precise effects, other than apoptosis, of p53 in lymphocytes.

## Conclusions

**Fig. 6:**
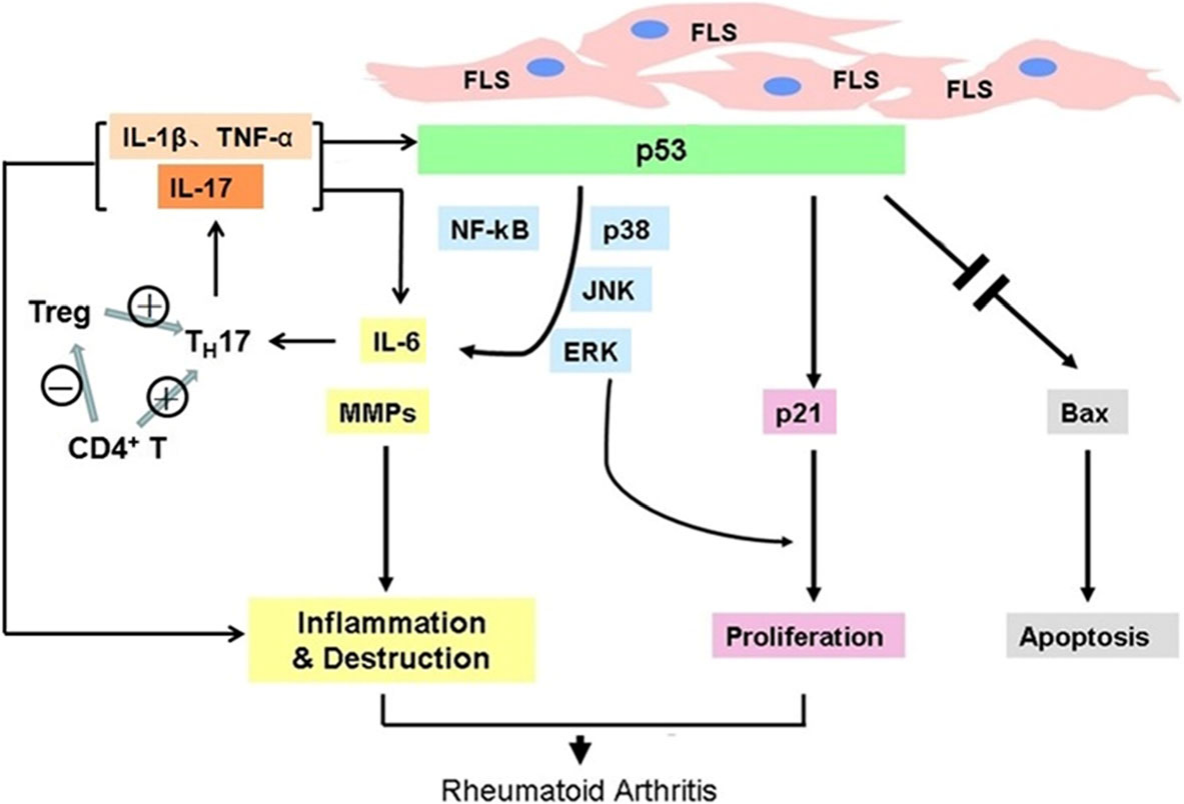
p53 plays a pivotal role in inhibiting synovial inflammation. The inflammation of the synovium in RA significantly inhibits p53 expression or leads to p53 dysfunction. In turn, suppressed p53 results in increased secretion of IL-6, which further suppresses p53, probably by modulating Th17 differentiation. *FLS* fibroblast-like synoviocytes, *IL* interleukin, *TNF* tumor necrosis factor, *TH17* IL-17 producing T helper, *Treg* regulatory T

In addition to its regulation of the cell cycle and apoptosis, p53 might play a crucial role in suppressing synovial inflammation by interaction with signal transduction pathways and regulating the production of inflammatory mediators to control RA (Fig. 6). Therefore, p53 may be a key player in the complicated network of synovial inflammation, proliferation, and apoptosis, making it a potential target for gene and biologic therapeutic strategies in RA.

## Abbreviations

Ad: Adenovirus
AIA: Adjuvant-induced arthritis
CD: Cluster of differentiation
CIA: Collagen-induced arthritis
DMEM: Dulbecco’s modified Eagle medium
DMSO: Dimethyl sulfoxide
DNA: Deoxyribonucleic acid
ELISA: Enzyme-linked immunosorbent assay
ERK: Extracellular signal-related kinase
FLS: Fibroblast-like synoviocytes
H&E: Hematoxylin and eosin
IgG: Immunoglobulin G
IL: Interleukin
IκB: Inhibitor of NF-κB
JNK: SAPK/c-Jun N-terminal protein kinase
MAPK: Mitogen-activated protein kinase
MMP: Matrix metalloproteinase
MOI: Multiplicity of infection
MTT: 3-(4,5-cimethylthiazol-2-yl)-2,5-diphenyl tetrazolium bromide
p38: p38 mitogen-activated protein kinase
PBS: Phosphate-buffered saline
PUMA: p53-upregulated modulator of apoptosis
PUMC: Peking Union Medical College
RA: Rheumatoid arthritis
RLU: Relative luciferase units
RNA: Ribonucleic acid
SEM: Standard error of the mean
TGF: Transforming growth factor
TH17: IL-17 producing T helper
TNF: Tumor necrosis factor
Treg: Regulatory T
TUNEL: Terminaldeoxynucleotidyl transferase dUTP nick end labeling
VCAM: Vascular cell adhesion molecule
